# The emotional challenges of conducting in‐depth research into significant health issues in health geography: reflections on emotional labour, fieldwork and life course

**DOI:** 10.1111/area.12347

**Published:** 2017-05-09

**Authors:** Sarah McGarrol

**Affiliations:** ^1^ Department of Public Health and Policy Health Protection Research Unit in Gastrointestinal Infection (NIHR) Farr Institute @ The Health eResearch Centre University of Liverpool Liverpool L69 3GL

**Keywords:** Scotland, life course, fieldwork, qualitative, emotional labour

## Abstract

Emotions are increasingly being recognised and integrated into human geography and it has been highlighted that focusing on the ‘interrelatedness’ of the research process is crucial. By contextualising fieldwork within the life course of the researcher, greater acknowledgement of the ‘emotional labour’ involved in fieldwork can be highlighted. The author reflects on the ‘emotional geographies’ of conducting PhD research into significant health issues with participants who had recently suffered a heart attack in Fife, Scotland. This paper reveals emotions involved in this kind of research, drawing on perspectives from participants as well as the researcher. The author also draws attention to, and reflects on, the lack of engagement with researcher's emotional labour within formal academic structures, such as research training and ethics application processes. Reflecting on fieldwork experiences from a distance, the author discusses the influence and impact of her emotional experiences of fieldwork. This paper contributes to work concerned with emotions and fieldwork in geography and asserts that greater importance and value needs to be given to this type of emotion work as embedded and situated within researchers’ life courses.

## Introduction

Bondi (2005) has stated that it is crucial and ethically imperative to take seriously people's emotions and emotions are increasingly being recognised and incorporated into human geography (Davidson *et al*. 2005, 2012). Within geographies of illness and disability, researchers have recognised and taken great care to represent the emotional experiences of those whom they study, in a variety of different ways (see for example Dyck 1999; Davidson and Smith 2003; Parr 1999, 2000; Milligan 1999). The purpose of representing emotions is to make sense of people's experiences, but also to assert that emotions matter (Davidson and Milligan 2004). Authors such as Bennett (2009) and Bondi (2003a, 2005, 2014b) have suggested that qualitative research is about meaning‐making and meanings are generated relationally. These cannot only be explained through the self, but through relationships with others, within the contexts of such interactions, such as fieldwork. It is therefore crucial to offer greater understanding of the importance of emotions in the research process. This must also include emotional experiences from the researcher's perspective. Widdowfield outlined that ‘not only does the researcher *affect* the research process, but they themselves are *affected* by this process’ (2000, 200). Therefore, it is important to provide narratives from researchers’ perspectives which not only acknowledge the importance of emotions, but situate the inter‐relatedness of (emotional) fieldwork experiences within researchers’ life courses.

## Theoretical concerns

Elder (1994) identified four dominant themes in the life‐course approach: the interplay of human lives and historical time, timing of lives, linked or interdependent lives and human agency in making choices (Hutchison 2010). The life‐course approach is inherently about the experience of time, as well as the interaction between individual biographies and associated socio‐cultural contexts, affected by historical and demographic trends (Worth 2009; Backett and Davison 1995). As such, looking to life‐course theory to frame emotions may be a way to conceptualise and present fieldwork experiences as interwoven into the biographical, historical and social time of the researcher's life course (Elder *et al*. 2003). Elder *et al*. (2003) also assert that social and professional lives are (differentially) affected by time and place during the life course. Fieldwork, then, ought not to be viewed as a distinct phase disconnected from other aspects of the researcher's personal and professional life, but as intertwined. Fieldwork cannot (and should not) be separated from the life course but rather contextualised and presented as inter‐linked.

Aligned with this is the importance of recognising the ‘emotional labour’ that can be involved in conducting qualitative research (Hochschild 1983; Phillips 1996). Emotional labour is defined as work that ‘involves feelings and may be contrasted with physical or task‐oriented labour’ (Hochschild 1983 in Hubbard *et al*. 2001, 121). The researcher's job, during the interactive process of interviewing, includes acts of ‘emotional labour’ whereby the researcher is required to be emotionally aware and sensitive to their own emotions, as well as those of the participants (Carroll 2013). There can be emotional risks for the researcher of conducting qualitative research particularly when researching sensitive topics. These emotional risks may affect researchers in different ways, at different times during their life course, with more novice researchers, potentially and inadvertently, neglecting the impact such research may have on them (Hubbard *et al*. 2001; Dickson‐Swift *et al*. 2007, 2008; Carroll 2013). Fieldwork also includes emotional connections with people *and* places (Davidson *et al*. 2005), which can include risks to the well‐being of the lone researcher. Within formal structures in the academy (for example, within research training, or ethical application procedures), there is a lack of attention to, and acknowledgement of, the emotional labour involved in fieldwork, and novice researchers may be unprepared for the level and extent of exposure to emotionally stressful situations (Sampson *et al*. 2008; Woodby *et al*. 2011).

In this paper, I present aspects of fieldwork conducted for my PhD research into men's and women's experiences of heart attack and recovery in socio‐economically contrasting areas in Fife, Scotland (McGarrol 2014). This PhD, funded by NHS Fife Managed Clinical Network for Coronary Heart Disease, was undertaken at the University of St Andrews, Fife, Scotland (2007–2014). The participants were 39 men and 11 women who had survived a heart attack. Interviews were conducted from March to July 2010.

The aim of focusing on my fieldwork experiences (interviewing) is to highlight why and how emotions matter in fieldwork. Contextualising these within my life course, I argue that emotions influenced and shaped fieldwork in complex ways, but that these experiences are embedded within my life course. Through reflecting on fieldwork some years later (post‐PhD), I argue that the emotional experiences generated during fieldwork continue to influence my life course in myriad interlinked and interactive ways.

## Setting up fieldwork: interviews

Before commencing fieldwork, the study was approved by both the School of Geography and Geosciences University Teaching and Ethics Committee (UTREC) and the East of Scotland Research Ethics Service, part of NHS Fife, NHS Forth Valley and NHS Tayside. The ethical application procedure included minimising risks, vulnerability and distress to the participants. Only cursory detail, however, was required about potential physical risks to the researcher, with risks affecting my psychological and emotional health and well‐being not considered in detail within either of the aforementioned formal ethical procedures (Dickson‐Swift *et al*. 2005, 2007, 2008). Within both applications, I was asked to outline the ‘risks to the researcher’. This is the paragraph which (at the time) I included in the forms:

The main risk to the researcher is that interviews are to be conducted in the homes of the participants. The reasons for choosing the home as the location for data collection are to minimise the inconvenience for the participants and to locate the data collection in the sociocultural context of the area where the participant lives. Interviews may be undertaken in areas unfamiliar to the researcher and/or after dark. In order to minimise the risk to the researcher, the researcher will inform both academic and NHS supervisors of the date, time and place of interviews on an ongoing basis via email and the secondary academic supervisor will act as a ‘buddy’ whom the researcher will text after completion of each interview in order for the researcher to be locatable at all times. Any details arising during the course of the interviews which prove distressing to the researcher will be dealt with through face to face or telephone supervision with the supervisors.

It is notable that there was little detailed attention given to emotional or psychological risks which could arise for the researcher undertaking fieldwork with participants in their homes. Indeed, postgraduate research training rarely considers in detail the health and well‐being of researchers (Bloor *et al*. 2007). There was no ‘lone worker policy’ that I was aware of. There was a lack of attention to, and little discussion with PhD supervisors about the emotional risks that this type of research might encompass, either before, during or after fieldwork. Preparing for, and commencing fieldwork, I did not acknowledge (or anticipate) the extent of the emotional and intellectual labour that was required to organise and conduct these interviews. I assumed that this was a normal part of the emotional management required during fieldwork. The feeling of fending for oneself during fieldwork was particularly acute (Emerald and Carpenter 2015a, 2015b; Blix and Wettergren 2015).

Laurier and Parr have stated that anxiety is ‘the classic interviewer's emotion’ (2000, 99), yet this anxiety preceded interviewing, and included worries around gaining ethical approvals, scheduling and pacing interviews (Hubbard *et al*. 2001). I planned to cluster interviews together geographically, in nearby locations on the same day. This was done to save time and limited research travel resources because my PhD funding was due to end in August 2010, within six months of starting fieldwork. Interviews were scheduled to take place at the convenience of participants at a time of their choosing. I was strategic in organising interviews and timings but getting to destinations (often unfamiliar) at appropriate times was challenging. Beale *et al*. (2004) have highlighted that it is best to avoid evening interviews to decrease researcher (and participant) fatigue, but at times this was unavoidable because some participants preferred evening interviews. In hindsight, organising multiple interviews on the same day was not always a sensible strategy, as lack of spacing between interviews did not provide enough time for ‘psychological or emotional recovery’ (Beale *et al*. 2004, 143). In addition, at the beginning of the fieldwork period, an unusually hard winter had gripped much of Eastern Scotland with snow, ice and frost. Combined with conducting research in many unfamiliar places, concerns with scheduling, apprehensions about travel arrangements due to weather conditions and the (un)suitability of my vehicle to cope with such conditions, anxiety and feelings of uncertainty were commonplace.

Referring back to my interview diary, I interviewed nine participants over three days in one week, in different locations across Fife. The practicalities of fieldwork were challenging. As fieldwork progressed, juggling worries about completing interviews and concerns about finances were coupled with the stresses of day‐to‐day journeying to and from home and multiple interview sites.

## Conducting the interviews

In addition to these practical concerns, I was also preoccupied with how I would be perceived by the participants in terms of my identity (professionalism, class, gender and age), which also generated anxieties (Madiega *et al*. 2013). At the time, I was a PhD student in my early 30s. I was older than the majority of PhD students within the same academic department. But I was younger than all the participants I interviewed, whose ages ranged from 44 to 81, and who were at quite different life‐course stages compared to me. Although I had previous interviewing experience, I did not have experience in interviewing participants who had recently experienced such a serious life event. I had not suffered a severe, life‐threatening condition, although I knew people who had. I did have a family member who was very ill during my fieldwork, which affected me greatly. These biographical, historical and social aspects of my life course at that time (Elder *et al*. 2003) undoubtedly influenced fieldwork interactions in overt and subtle ways.

My social and professional position was perceived in a number of different ways by participants and impacted, at times, on the building of rapport and empathy. Bondi (2003b) has outlined that fieldwork is inherently uncertain and it is not known whether the research relationship will result in identification or empathic understanding. Many of the participants were naturally curious about the research and curious about me. Although participants and I had chatted on the phone to organise the interview, my arrival on their doorstep was our first face‐to‐face contact. These first impressions were important and sometimes anxiety‐provoking, as I was unsure how well the participants would be feeling that day, what their living conditions would be like or who else might be with them.

A number of participants wondered whether I was ‘posh’ because I was from the University of St Andrews. St Andrews is a university with a long‐standing reputation for attracting private or independent school students and references to this ‘posh’ reputation and speculation about my ‘posh‐ness’ were mentioned. A particular memory of an early interview was with Jason,^1^ aged 48. He was guarded with me from the outset and I recall not feeling particularly comfortable in his home or particularly empathetic towards him initially. He made a comment about me based on his perception of me, e.g. ‘posh and snobby’. This felt unfair and personal, as I would not have identified myself as either ‘posh’ or ‘snobby’ despite studying at the University of St Andrews.

While exploring social and health inequalities, I asked him if he thought where he lived was unhealthy compared with other places in Fife. He responded, ‘Well, you know this postcode, healthcare thing. Well this is a shithole postcode so nobody bothers.’ I asked him to elaborate. ‘Well, it's like I'm saying, if *you* go to the hospital and you come from a nice area, then you'll get treated no bother, but if you come from here, you don't. Well you're bound to know that yourself. If you go to the doctors, you know, you go to Bupa,^2^ you don't go to the National Health Service.’ I replied saying I did ‘go to the NHS ‘cause I'm a student’. Jason replied, ‘Aye, but you won't be for long.’

Others thought I was an ‘NHS person’. For example, Reg, 74, queried ‘I don't have trouble with my heart. I think I had a stroke. I've got a box of pills through there. I don't know what they do. Do you? I feel like stopping them all together and starting from scratch.’ Others saw a ‘young lassie’^3^ and heard my accent, asking if I was ‘local’ or ‘fae Dundee’.^4^^,^ 5

These exchanges served as examples of participants’ perceptions of our differing social and health positions.

It was clear that the heart attack was a major life‐course event for the participants. Descriptions of the heart attack as a traumatic, shocking and disruptive occurrence were commonplace within the interviews (Kristofferzon *et al*. 2007; Allison and Campbell 2009). The sudden onset and surprising nature of a serious condition can ‘assault the body and threaten the integrity of self’ (Charmaz 1995, 657). Individuals can struggle to make sense of their illness and come to terms with what has happened. The notion that the self is in crisis through the experience of a chronic condition has been outlined as ‘biographical disruption’ (Bury 1982; Williams 1996, 2000). Taken for granted assumptions and behaviours can be called into question. In attempting to explain what has occurred, the individual has to reformulate their biography and sense of self around the chronic illness experience and practical responses to the disruption. This involves mobilising ‘physical as well as social, temporal as well as financial, medical as well as cultural’ resources (Williams 2000, 43). Gavin, 60, outlined that ‘I've still got this thing in my mind that I've had this heart attack and it could just hit again.’ The after‐effects of the heart attack raised varying emotional reactions for participants and thoughts about identity, mortality and the disruptive effect of the heart attack, including accounts of pain and suffering, as well as reflections on their past and future, were frequently discussed. Laurie, 59, stated that his heart attack was

Overwhelming. You can't describe it. It's like death. That's what this feeling is like and I find it difficult to believe that because I've had a heart attack, I'm not going to have another one. It's in my mind that I won't see the year out.

An acute event, like a heart attack, juxtaposes the former self (before heart attack) with the new impacted self (after heart attack). This identity re‐evaluation is an on‐going process. The consequences of the heart attack can impact and upset daily life in practical, physical, social and psychological ways (from mundane tasks, through work, to social relations and other aspects besides). For example, Elspeth, 73, stated that

From now on I don't see myself as having much of a life now to be quite honest with you. I think I will get through this and I've never been one that felt sorry for myself. I've never, ever been like this, but there's too much going on at the moment for me and I'm not coping with it awful great. I'm hoping to get back to what I was. Maybe I'll never get back to what I was but I just hope I will get back to being able to do things for myself and enjoy myself, even at my age.

Worries about the future and concerns over ‘getting back to normal’ were commonplace, suggesting that life‐course trajectories were disrupted and in flux due to the heart attack (life event). When I interviewed Janet, 50, she became quite upset. ‘I've had to change a lot of things but I'm frightened’ (starts crying). I replied, ‘Oh, I'm sorry. Please don't get upset.’ Janet continued to speak through tears, saying, ‘I'm alright. I keep thinking, stupid bitch Janet. Why did you not stop smoking all those years ago? And I think that's what makes me panic. When I get short of breath I think, ‘Oh Christ!’

The consequences of a heart attack can be differentially experienced depending on a variety of factors and how these inter‐relate with the individual's life course. The heart‐attack experiences interlinked with other aspects of participants’ life courses and although all the participants had experienced a heart attack, their age, gender and social position affected these experiences in different ways. Participants’ life‐course trajectories were unquestionably affected in complex ways after their heart‐attack experiences and participants gave accounts suggesting that they too were ‘engaged in emotion work in an attempt to maintain their authentic selves in the context of life course disruption’ (Exley and Letherby 2001, 125).

## After the interviews

Developing and maintaining rapport and being empathetic involved ‘continuous emotion work’ (Blix and Wettergren 2015, 691) and attending to these often emotional accounts and the accumulation of these stories over ‘time‐space’ necessitated emotional management (Blix and Wettergren 2015). The emotional variation within participant accounts demanded a high degree of sensitivity to their psychological and emotional needs (Minichiello *et al*. 2008). At times I felt confusion in the role I should take, or the role that participants expected or wanted me to take (researcher, counsellor, ‘NHS person’). I had doubts about my proficiency at maintaining a professional ‘distance’ when personal disclosures were made and I often reflected on why some research situations called for emotional admissions, some of which felt exposing, while others did not (Madiega *et al*. 2013). These formed a crucial part of the fieldwork encounters and required on‐going inter‐personal negotiations, highlighting the complex relational and emotional skills required in qualitative research (Bondi 2014a). Reflecting on my own emotional aptitude in managing and maintaining my emotions, as the fieldwork progressed, these depleted in a number of ways. Listening to accounts of participants’ worries and uncertainties echoed my own uncertainties (albeit in different ways). Doubts crept in about my ability (and desire) to empathise and identify with their experiences. The emotional labour involved in conducting the interviews grew burdensome, and the interviews began to exert an emotional toll (Bondi 2003a, 2005, 2014b; Hubbard *et al*. 2001; Emerald and Carpenter 2015a).

Combined with academic concerns about ensuring the fieldwork was on schedule, if I was doing it right and if it was of sufficient quality, anxieties and feelings of insecurity rumbled disquietly alongside other personal emotional highs and lows during this period. These inter‐related aspects of my social and professional life began to blur and worrying about the ‘bigger’ picture of completing fieldwork, running out of time and concerns about finances, coupled with my emotional journeying through fieldwork, left me with an emotional hangover. These hangovers persisted in different ways with emotional and psychological feelings of disquiet and uneasiness lingering after the interview(s) had concluded, but also these segued into later stages of the research process. I was affected emotionally by many of the accounts. I often felt drained, sad, guilty, lonely, powerless and exhausted after the days, weeks and months of conducting interviews (Dickson‐Swift *et al*. 2008, 2009; Punch 2012). By the time fieldwork was concluded in July 2010, the accounts from the ‘wounded story tellers’ (Frank 1995) had resulted in a wounded researcher.

Previous research has outlined that feelings of guilt can occur after the research relationships have come to an end (Burr 1995; Cannon 1992) and I felt this acutely as I had learned that three of the participants whom I had interviewed had since passed away. I felt an urgency to get their stories out, yet I was struggling with how to make sense of the experiences I had heard, both professionally and personally (Emerald and Carpenter 2015a). The emotional labour involved in this research was significant and had implications for other parts of the research process. My funding terminated shortly after fieldwork and I took a leave of absence from my PhD studies to pursue a research position at another university. These situations impacted on PhD completion, taking another three years to submit. Regretfully, in the submitted thesis, little attention was paid to the emotional nature of this kind of research, or my direct emotional fieldwork experiences.

## Conclusions

This paper has presented a number of reflections highlighting the emotional labour involved in conducting research into the geographies of men's and women's heart‐attack experiences. This paper has also framed and situated these emotional experiences within the biographical, historical and social time that they are interwoven into, in this case during PhD fieldwork, through reflecting on these experiences a number of years later (Elder *et al*. 2003). I have highlighted the importance, and necessity, of giving greater attention and recognition to the emotional labour and emotional experiences involved during the research process, particularly fieldwork. My experiences are shown as multi‐dimensional and inter‐related with other personal and professional aspects of my life course, and are also interwoven with the life‐course experiences of the participants after a significant health issue – a heart attack. Despite the life‐course differences between me and the participants, I attempted to understand participants’ experiences empathetically and I believe I did, as evidenced by my own emotional exhaustion, during and after fieldwork, generated perhaps by an ‘over‐identification with interviewees’ (Evans 2012). This exhaustion was exacerbated by the insufficient acknowledgement of, planning for or engagement with the emotional labour required in conducting this type of research.

There is a continuing lack of attention paid to researchers’ emotional labour or emotional well‐being within the academy. Emotions are key to many aspects of research, including fieldwork, but the emotional nature of research and the emotional risks to the researcher are often hidden, omitted or undervalued (Hubbard *et al*. 2001). It is my contention that ‘bringing one's [emotional] self into the research process’ (Widdowfield 2000, 199) is important for contextualising research within and to the life course of the researcher and highlighting the ‘complex nature of emotion work in fieldwork’ can also be considered to be work on the self (Exley and Letherby 2001, 129). This is an on‐going process (Worth 2009). In writing this paper, seven years after conducting fieldwork, it illustrates that I am ‘still emotionally engaged’ with these experiences (Bondi 2013, 14), and that these experiences have since shaped and influenced my personal and professional identity. In this paper, framing fieldwork as intertwined within my life course highlights the emotional demands and complexities involved in fieldwork experiences. They are linearly presented, from setting up and conducting interviews, through to experiences post‐fieldwork. However, these fieldwork experiences did not start or end at certain time‐points, but have endured as part of my life course.

## Notes

1 Participants’ names are pseudonyms.

2 BUPA: private healthcare company whose headquarters are in the United Kingdom.

3 ‘Lassie’ (Scots): meaning ‘girl’.

4 ‘Fae Dundee’ (Scots): meaning ‘from Dundee’.

5 During my PhD I lived in Dundee, Scotland's fourth largest city, which is located on the East of Scotland, situated on the Firth of Tay which overlooks North Fife.
